# Supplementary material for: Getting the brain into gear: An online study investigating cognitive reserve and word-finding abilities in healthy ageing
Source: PLoS One. 2023 Apr 20;18(4):e0280566. doi: 10.1371/journal.pone.0280566 (PMC10118119; doi:10.1371/journal.pone.0280566)
Supplement: Attachment — Submitted filename: 19122022_PLOSONE_ResponseToReviewers_Final.docx [file pone.0280566.s001.docx]

**Reviewers’ Comments to the Authors:**

**Reviewer 1**

Major

1. The truncation of the data seems odd to me (and not without consequences, see e.g., Jeff Miller's work), especially in the context of studying populations that are likely to start deviating more (i.e., the older group), a deviation that is relevant. But even if the authors choose to trim the data, it would be important to be explicit about how the 2.5 SD was calculated: over participants, within participants, per task, etc? This is made explicit in line 263 but it would be important to clarify. In any case, especially in the older group, more extreme values could be relevant for the research question rather than outliers. If the authors choose to keep the procedure of trimming the data, I would suggest they do mention the potential issues.

**Author response:** Thank you for raising this issue. We agree that trimming the data may result in a loss of variance that could provide important insights. However, there are studies that report greater bias and an increased probability of Type-II errors when data is not trimmed (including reaction time data; (Berger & Kiefer, 2021). Berger & Kiefer (2021) reported that the least bias was found when using *z-*scores to trim the data. Furthermore, we decided to pre-register our choice of removing outliers, which is recommended by Berger & Kiefer to reduce additional bias. We agree that readers should be informed about the potential issues of trimming the data and have incorporated information on this in the Discussion on page 28. We also clarified in the methods section how the 2.5 SD was calculated (i.e., within each age group) and that the decision of trimming the data was pre-registered.

**Page 14, Paragraph 2, Lines 289-291:** *“Following the preregistration, the data was trimmed using z-scores to reduce the probability of Type-II errors (67). The dependent variables were transformed into z-scores within each age-group and outliers were identified as being above and below 2.5 SD. That is, …”*

**Page 28, Paragraph 2, Lines 582-594:** *“In the current study, we transformed the picture-naming reaction times and the number of correctly produced words on the verbal fluency tasks into z-scores within each participant group. We then classified any score above or below 2.5 SD as an outlier, which was subsequently removed. It is theoretically possible that trimming data, including the above-mentioned method, can lead to distortions of the data set (84,85). However, recent research suggests that not excluding outliers leads to a greater negative bias (67). That is, the probability of Type-II errors increases when the data is not trimmed. Using the z-score method mentioned in this study leads to the smallest bias. With increasing age, standard deviations in cognitive tasks increase, meaning more variance within older than younger samples. Hence, trimming the data, especially in older adults, may lead to masking of deviation effects. In the current study, we chose to trim the data within each of the age groups, reducing the chance of masking such deviations. In addition, it is likely that trimming the data using z-scores has led to lower bias than not trimming the data at all (see 83).”*

References:

Berger, A., & Kiefer, M. (2021). Comparison of Different Response Time Outlier Exclusion Methods: A Simulation Study. *Frontiers in Psychology*, *12*. <https://www.frontiersin.org/articles/10.3389/fpsyg.2021.675558>

2. lines 429-435: The discussion on cognitive resources in ageing is put a bit simplistically in my view. The statement "If these theories are correct [...]" (line 429) is true critically under the assumption that the measures employed are good measures for capturing those constructs. Unfortunately, the state of cognitive sciences/neuropsychology is such that this assumption barely ever holds in its strong form. I would consider making this hidden premise explicit in the discussion.

**Author response:** Thank you for bringing this hidden premise under our attention. We have made this explicit in the discussion on page 24

**Page 24, Paragraph 1, Lines 470-476: “***If the above-mentioned theories are correct, an age-related reduction in cognitive resources would explain the variance in the reaction times for action and object naming in the current study. Note this point holds true only under the assumption that the measures employed are good measures for capturing the aforementioned constructs. However, pure measures of complex psychological constructs, such as cognitive processing speed, are difficult to capture because measures can be influenced by multiple factors in addition to the construct itself (79).”*

3. Related to the above: a cautionary note could be given on the underlying assumption of a causal link between CR --> cognitive functions. This remains a tricky issue: Can one really know for sure that it's CR that affects cognitive functioning, rather than any other causal relationship (that one did not measure)?

**Author response:** Thank you bringing this point to our attention. It is well known that many factors can influence cognitive ageing. For example, hearing loss can impact our cognitive abilities (Slade et al., 2020), as well as smoking and high blood pressure (Sebastiani et al., 2020), factors that haven’t been taken into account in the current study. We commented on this issue in the discussion and included suggestions for future research on page 25.

**Page 25, Paragraph 2, Lines 511-518: “***A cautionary note should be given as it is currently unclear whether there is a causal link between CR and cognitive functioning. Several factors can influence cognitive ageing that have not been measured here, such as hearing loss (81), smoking and high blood pressure (82), to name a few. Such factors have not been considered in the current study but could in theory have exerted influence on the relationship between CR and word-finding abilities. Longitudinal studies that control for such factors and investigate the causal role of CR interventions on cognition would provide critical information regarding the causal link between CR and cognition.”*

4. page 7, lines 160-163: This is unclear, at least at this point, the reader has no way of knowing if participants were tested twice, once pre and once during, or why in any case there would be one measurement preceding and one coinciding with the pandemic. Moreover, this issue is not clarified at any point in the manuscript.

**Author response:** We agree with the reviewer that the manuscript would benefit from clarifying how often and when participants were tested. The study was conducted during the pandemic and as such, it was not possible to have collected data before the COVID-19 pandemic. Hence, we took a retrospective baseline measurement. We clarified this by explaining how we measured cognitive reserve for the period preceding and coinciding with the COVID-19 pandemic on page 7. In addition, we now explicitly state in the procedure section that participants were only tested once:

**Page 7, Paragraph 3, Lines 168-173:** *“The CR questionnaire was split into two parts. The first part required participants to report the frequency of their activities for the period preceding the COVID-19 pandemic. That is, the participants were asked to report the frequency of their activities retrospectively. The second part of the CR questionnaire required participants to report the frequency of their activities for the period coinciding with the COVID-19 pandemic.”*

**Page 13, Paragraph 2, Lines 272-273** *“Each participant completed the CR questionnaires and the behavioural tasks only once.”*

**Page 22, Paragraph 1, Lines 430-432:** *“It is possible that social isolation due to the COVID-19 pandemic led to changes in CR scores, which could impact current word-finding ability.* *Table 1 provides the descriptive statistics of the measures used to compute CR, for the period preceding and coinciding with the COVID-19 pandemic separately.”*

Minor (in no particular order)

5. lines 253-254: "Multiple linear regression was conducted on the verbal fluency data as there were no repeated measures". In a way, there are since there were multiple prompts per fluency task. Is there a reason why the authors chose to work with averaged scores for this task (cf. picture naming)?

**Author response:** Thank you for raising this point. We chose to create a composite score for each of the three verbal fluency tasks to reduce the number of multiple comparisons. We clarified this choice in the manuscript on page 11:

**Page 11, Paragraph 2, Lines 226-228:** *“Composite scores for each of the three verbal fluency tasks were created to reduce the number of multiple comparisons.”*

6. were objects and actions also matched for word length? Although not a critical variable (e.g., Alario et al. 2004), it could be helpful for the reader to provide that information

**Author response:** Objects and actions were not matched for word length. We added this information on page 10:

**Page 10, Paragraph 2, Lines 190-193:** *“Action and object words were not matched for word length. However, this should not affect our results as word length is unlikely to influence naming speed* *(50). In addition, we did not directly compare naming speed between object and action naming.”*

7. lines 259-262: I do not understand why the percentage of removed data differs depending on whether one is looking at number of produced words or their frequency. Shouldn't they be the same, i.e., data is removed and from that one calculates the number of produced words and their frequency?

**Author response:** Thank you for this point. To remove outliers using *z-*scores, we followed the procedure of previous studies (e.g., Berger & Kiefer, 2021). For this, we need to convert the outcome variables first into *z-*scores before we can remove the data. Hence, we need to remove the data after calculating the number of produced words and their frequency, because we need their *z-*scores in order to be able to remove data below/above 2 SD.

8. lines 283-284: "random effects for Trial (i.e., variance caused by between-item variability)", why trial and not item ID? In my understanding, the item ID will capture the between-item variability, trial will not; instead, it will capture variability due to passing of time (given that items were presented in randomised fashion).

**Author response:** We agree with the reviewer that the random effect should be termed item ID and not trial ID. Item ID was indeed what we included as the random effect in the analysis. For clarification, we have changed the name “*Trial ID*” to “*Item ID*” throughout the manuscript. In addition, we decided to change “*Subject ID”* to “*Participant ID*” to enhance clarity. This was done on pages 15 and 18, and in tables 2 to 5.

9. 2.2.2: Please, clarify if objects and actions were presented in a blocked manner or fully randomised. Given the sentence in line 175, "Both the object and action picture-naming task", I assume they were blocked but it would be helpful to have this information explicit.

**Author response:** Indeed, the object and action naming tasks were presented in a blocked manner. We added this information on page 10.

**Page 10, Paragraph 3, Lines 199-200:** *“The picture naming tasks were presented in a blocked manner. That is, participants first completed the object naming task after which they completed the action naming task.”*

10. page 3, "Word-finding difficulties can extend beyond the difficulties associated with healthy ageing in dementia (8,9)." Is there a phrasing issue here "healthy ageing in dementia"?

**Author response:** Thank you for indicating the phrasing issue in this sentence. We rephrased the sentence on page 3 so that healthy ageing and dementia are not linked anymore:

**Page 3, Paragraph 2, Lines 70-71:** *“… associated with healthy ageing, which could signal the onset of dementia (8,9)”*

11. page 7, lines 167-168: are the number in parentheses SD? Please, indicate.

**Author response:** We clarified that the words in parentheses represent standard deviations on page 10:

**Page 10, Paragraph 2, Lines 187-18189:** *“… frequency (actions: M =3.3, SD = 1.8; objects: M = 3.1, SD = 1.7), age of acquisition of the words (actions: M = 5.2, SD = 1.7; objects: M = 4.8, SD = 1.4), and …”*

12. lines 247-248: "reaction time of correct trials only and 2) accuracy for object and action naming separately": This suggests the RT data was collapsed for naming task whereas the accuracy data was not. I don't think that was the case but maybe the wording can be made clearer?

**Author response:** Both reaction time and accuracy were analysed separately for object and action naming. This is clarified on page 13:

**Page 13, Paragraph 3, Lines 279-290:** *“… 1) reaction time of correct trials for object and action naming separately and 2) accuracy …”*

13. First paragraph of Limitations: I do not see how this is a limitation of the study. Rather, I would consider it a strength that the authors went further than just using years of education as a measure for CR.

**Author response**: Thank you for pointing out this as a strength of the study. We moved this paragraph out of the limitations into the discussion section, on page 27, paragraph 2, lines 561-566.

**Reviewer 2**

2.1. Major comments:

1. Introduction: The authors discuss brain networks and recruitment of additional brain networks, as ‘cognitive reserve’. This is controversial as there are separate constructs for ‘cognitive reserve’ and ‘brain reserve’. Can the authors please discuss the distinction between these concepts?

**Author response**: We agree with the reviewer that brain reserve and cognitive reserve are two different concepts. According to cognitive reserve literature (Gonzalez-Burgos et al., 2020; Stern, 2002; Stern et al., 2020), lifestyle choices and lifetime experiences can increase brain flexibility and neural efficiency. Individuals with greater ‘brain reserve’, i.e., more neurons, are thought to be better able to tolerate neurological attrition before it becomes pathophysiological. Indeed, through ‘brain maintenance’ some individuals maintain their brains more so than others, i.e., through life experience and genetics. To increase the readers’ understanding of the differences between these concepts, we added information on brain reserve in the introduction. Also, we clarified that cognitive reserve could, among others, facilitate the recruitment of additional brain networks.

**Page 3, Paragraph 1, Lines 56-57:** *“Greater CR can enable the use of …”* and **Lines 59-67:** *“Brain reserve is another concept often discussed in conjunction with CR.* *Brain reserve reflects the brain’s anatomical resources, such as number of neurons, and individuals with greater ‘brain reserve’ (i.e., more neurons) are thought to be better able to tolerate neurological attrition before it becomes pathophysiological (6). Indeed, through ‘brain maintenance’ some individuals maintain their brains more so than others, for example, through life experience and genetics. Such brain maintenance can therefore increase brain reserve (6). Brain reserve differs from CR in that CR explains individual differences in the ability to cope with cognitive difficulties through differences in lifestyle choices and life experiences.”*

2. Method: The power calculation could be described more clearly. How many people were required to achieve 80% power and .05 alpha at a large effect size? What is the level required for a large effect size?

**Author response**: Thank you for this point of clarification. We added this information to the description of the power calculation on page 6:

**Page 6, Paragraph 2, Lines 139-140:** *“… a large effect size (Cohen’s f = 0.40). This resulted in a sample size of 30 participants per age group.”*

3. Method- Participants: Given how common anxiety & depression are throughout life, it is surprising that no participants reported history of or current psychiatric disorders. Were participants asked about severe conditions like schizophrenia and bipolar or more common conditions such as anxiety or mood disorders?

**Author response**: Thank you for raising this issue. All participants were pre-screened as to whether they were suffering from psychiatric disorders, such as bipolar or schizophrenia. Hence, no participants were suffering from such conditions. Depression and mood were assessed using the Beck Depression Inventory-II. Participants above a score of 19 were considered as experiencing depressive symptoms and were excluded from further participation. This led to an exclusion of 14 participants due to depressive symptoms. We clarified these points in the participants’ section:

**Page 6, Paragraph 3, Lines 145-146:** *“… current neurological (e.g., stroke or epilepsy), psychiatric (e.g., schizophrenia or bipolar), …”*

**Page 7, Paragraph 1, Lines 148-149:** *“… and, hence, did not suffer from depression.”*

4. Method – Participants: The sample is not described in detail. What were their demographic details, such as ethnicity, and also education and time spent doing leisure activities (the variables used to calculate CR)?

**Author response**: We agree with the reviewer that the sample could be described in more detail. Participants were not asked to report their ethnicity. We added descriptive statistics on years of education, occupational attainment, and the frequency of participation in leisure activities in Table 1.

**Page 7, Paragraph 1, Lines 151-152:** *“Years of education, occupational score, and frequency of leisure activity can be found in Table 1.”*

5. Method- Participants: Why were only monolingual British English speakers chosen? The unrepresentativeness of the sample needs to be explicitly acknowledged in the Abstract and the Discussion.

**Author response**: In the current study, we decided to only include monolingual speakers of British English in order to control the variance in the data, as research indicates that bi- or multilingual people may be slower in word-finding in both their native and non-native language, compared to monolingual speakers. We completely agree this leads to a biased, unrepresentative sample, and suggest that future studies should investigate CR and bi/multilingualism. We clarified this point in the method’s section on page 6. Furthermore, we also acknowledged this issue in the abstract and limitation section.

**Page 6, Paragraph 2, Lines 133-135: “***To control for variance in word-finding speed between monolinguals and bi-/multilinguals (32), only monolingual speakers of British English were included in this study.”*

**Abstract, Line 33:** *“All participants were right-handed, monolingual speakers of British English.”*

**Page 28, Paragraph 1, Lines 576-582:** *“Furthermore, the current study was restricted to a sample of monolingual speakers of British English to reduce the variance in word-finding speed, which can be affected by language background (32). Hence, the results of this study cannot be generalised to multilingual speakers. Future studies should investigate this question using groups of speakers with varied language backgrounds to expand on the relationships between CR and word-finding abilities across the lifespan. Such future research will increase our understanding of the effects observed in the current study, which has a restricted sample.”*

6. Method: Please also provide descriptive statistics for the various measures used for the sample, as well as the internal consistency of the measures in the current sample.

**Author response**: Please, see the response to Reviewer 2, Comment 4. We also reported Cronbach’s Alpha to report the internal consistency of the measures on page 6. Since Cronbach’s Alpha was not very high, we discussed the implications in the discussion section on page 29:

**Page 9, Paragraph 2, Lines 174-176: “***Internal consistency was calculated using Cronbach’s Alpha (47) and was α = .59 for all items used to compute CR.”*

**Page 29, Paragraph 3, Lines 602-613:** *“The internal consistency of the CR questionnaire was poor (α =.59), indicating that the items in the questionnaire are multidimensional (87). The low internal consistency score could be due to few questionnaire items or because they do not measure the same underlying construct. CR is a concept that is believed to be built up by lifestyle choices and lifetime experiences, such as physical activity and education (6). Hence, CR in itself is a multidimensional construct and this multidimensionality might be reflected through the low Cronbach Alpha score. However, there is currently no consensus as to what lifestyle choices and lifetime experiences must be included to compute an individual's CR level (for a discussion, please see (80). To better capture CR, the current study included a range of CR proxies, including years of education, frequency of cognitively stimulating activities, and physical activities. Hence, the low Cronbach Alpha score may be a trade-off of using a more comprehensive CR measure.”*

7. Method: How was CR calculated? Were certain activities given more weight in the calculation? The method is vaguely described in the paper, but could be clearer.

**Author response:** Thank you for your suggestion for clarity. The different measures of CR were equally weighted when computing CR. We clarified this on page 8:

**Page 10, Paragraph 1, Lines 180-182** *“By averaging over the z-scores, education, occupational attainment, and general and physical activities were given equal weight when computing CR.”*

8. Method: Where does the information about before and during COVID come from? Were the participants asked questions at multiple time points or was the questionnaire structured to ask about before and during COVID?

**Author response:** We agree with the reviewer that the way we obtained the measures for the period preceding and coinciding with COVID-19 could be clarified. Data were collected only once. Please, see Reviewer 1, Comment 4 for further information.

9. Method: The tasks used for semantic and action Verbal Fluency and the letters for Letter Fluency (S, M, P instead of F, A, S) appear to be novel. Was there any prior validation study conducted on the appropriateness of these materials?

**Author response:** Thank you for pointing this out. The prompts for the verbal fluency tasks were adopted from previous studies. These previous studies validated the appropriateness of these prompts. We added this information on page 11:

**Page 11, Paragraph 2, Lines 222-224:** *“The verbal fluency prompts were based on previous studies, which also have validated the use of these prompts (20,21,52,53).”*

10. Method: Inhibitory control, working memory, and cognitive processing speed tasks were adapted for an online format and normed within each age group. There were, however, only 30 people in each age category. This is not a representative sample for creating age group norms. Is there any prior research investigating the reliability and validity of the online versions of these tests? Were scores adjusted for sex or level of education?

**Author response:** The scores for general cognitive processing were not adjusted for sex or level of education. Education was considered when calculating the CR composite score. Scores for the general cognitive processing tasks were not adjusted for sex or level of education. However, the variable was added as a covariate to the model and was not a main variable of interest.. Computerised control tasks have been used and validated before. For example, the Cambridge Neuropsychological Test Automated Battery have implemented and validated these three tasks in their task battery (Robbins et al., 1994). We added this information in the method’s section. Hence, we believe that the online versions of the general cognitive processing tasks have been proven reliable and valid.

**Page 13, Paragraph 1, Lines 257-260:** *“Computerised versions of these three control tasks are widely used and even implemented in and validated for cognitive testing batteries such as the Cambridge Neuropsychological Test Automated Battery (CANTAB; 59).”*

11. Statistical analysis: Please provide a reference for the “Nelder Mead” optimiser method.

**Author response:** We now added the reference to the analysis’ section.

12. Results: Why was depression measured (BDI-II) and then not mentioned again in the manuscript? Did the authors control for depressive symptoms in their analyses?

**Author response:** Thank you for this point of clarification. All participants were pre-screened for depression using the BDI-II. Any participants with depressive symptoms were excluded from the analyses. Hence, there was no need to control for depressive symptoms. Please, also see the response to Reviewer 2, Comment 3.

13. Discussion: The authors state that middle-aged older adults might draw on CR for word-finding, but older adults might use other cognitive processes. Can you please explain what these processes might be?

**Author response:** We agree this phrase could benefit from additional explanation. Instead of CR, older adults might rely more upon more general cognitive processing abilities, such as cognitive processing speed, working memory, or vocabulary (Cansino et al., 2020). We added this explanation on page 25:

**Page 25, Paragraph 2, Line 510:** *“… cognitive processes, such as cognitive processing speed, working memory, or vocabulary, that …”*

14. Discussion: The authors mention interventions to promote CR in later life. Can you please expand on what are some promising interventions to do so?

**Author response:** Thank you for this suggestion. We added examples of interventions on page 30:

**Page 31, Paragraph 1, Lines 648-650:** *“Such interventions can include cognitive training, education later in life, and promoting healthy lifestyle choices throughout the lifespan (for a discussion on interventions, please see 80).”*

2.2. Minor comments:

15. When reported standard deviations, please use “SD = value” instead of “SD value” in the Results

**Author response:** We now used SD = Value throughout the manuscript.

16. Please italicise statistical symbols (β, SD)

**Author response:** We have now italicised all statistical symbols throughout the manuscript.

17. Discussion, line 467-468: The grammar is odd in the first sentence of this paragraph. Can you please rephrase this?

**Author response:** Thank you for pointing out the grammar in this sentence. We have rephrased the sentence to remove any unusual and ungrammatical phrases:

**Page 25, Paragraph 4, Lines 519-520:** *“Furthermore, we observed that older adults experienced greater word-finding difficulties relative to both younger and middle-aged adults.”*
